# Photocatalytic and Photo-Fenton Catalytic Degradation Activities of Z-Scheme Ag_2_S/BiFeO_3_ Heterojunction Composites under Visible-Light Irradiation

**DOI:** 10.3390/nano9030399

**Published:** 2019-03-09

**Authors:** Lijing Di, Hua Yang, Tao Xian, Xueqin Liu, Xiujuan Chen

**Affiliations:** 1 State Key Laboratory of Advanced Processing and Recycling of Non-ferrous Metals, Lanzhou University of Technology, Lanzhou 730050, China; woshidilijing99@163.com (L.D.); chenxj@lut.cn (X.C.); 2 College of Physics and Electronic Information Engineering, Qinghai Normal University, Xining 810008, China; 3 School of Science, Chongqing University of Technology, Chongqing 4000054, China; xqliu@cqut.edu.cn

**Keywords:** polyhedral BiFeO_3_ particles, Ag_2_S nanoparticles, Z-scheme Ag_2_S/BiFeO_3_ heterojunction, photocatalysis, photo-Fenton catalysis

## Abstract

Z-scheme Ag_2_S/BiFeO_3_ heterojunction composites were successfully prepared through a precipitation method. The morphology and microstructure characterization demonstrate that Ag_2_S nanoparticles (30–50 nm) are well-decorated on the surfaces of polyhedral BiFeO_3_ particles (500–800 nm) to form Ag_2_S/BiFeO_3_ heterojunctions. The photocatalytic and photo-Fenton catalytic activities of the as-derived Ag_2_S/BiFeO_3_ heterojunction composites were evaluated by the degradation of methyl orange (MO) under visible-light irradiation. The photocatalytic result indicates that the Ag_2_S/BiFeO_3_ composites exhibit much improved photocatalytic activities when compared with bare Ag_2_S and BiFeO_3_. The optimum composite sample was observed to be 15% Ag_2_S/BiFeO_3_ with an Ag_2_S mass fraction of 15%. Furthermore, the addition of H_2_O_2_ can further enhance the dye degradation efficiency, which is due to the synergistic effects of photo- and Fenton catalysis. The results of photoelectrochemical and photoluminescence measurements suggest a greater separation of the photoexcited electron/hole pairs in the Ag_2_S/BiFeO_3_ composites. According to the active species trapping experiments, the photocatalytic and photo-Fenton catalytic mechanisms of the Ag_2_S/BiFeO_3_ composites were proposed and discussed.

## 1. Introduction

Wastewater containing organic dyes or pigments has caused serious damage to environment and human health in recent times. Moreover, most of the organic pollutants are toxic, non-biodegradable, and are difficult to mineralize under natural conditions [1]. Several wastewater treatment routes have been used to eliminate organic pollutants from wastewater [2,3,4,5,6,7,8,9,10]. Compared with other elimination methods, advanced oxidation processes (AOPs) are demonstrated to be attractive methods for the efficient elimination of organic contaminants. In these processes, strong oxidizing radical species such as hydroxyl (•OH) radicals can be generated; they degrade most organic pollutants into innocuous byproducts [8,9]. Among various AOPs, photocatalytic and photo-Fenton-like catalytic processes have attracted remarkable attention for the decomposition of dyes, owing to their low cost, mild reaction conditions, and easy operation procedures [11,12,13,14,15,16,17,18,19]. The photocatalysis process involves the generation of electron (e^−^)-hole (h^+^) pairs under suitable light irradiation, the transformation of photoexcited charges to the surface of photocatalyst, redox reactions of the charges with chemical species to form active species, and the degradation of pollutants by the attack of active species. The photo-Fenton-like catalytic process is based on the traditional Fenton process, and light irradiation. During the Fenton process, •OH radicals can be derived from the reaction of the Fenton reagent [16,17,18,19]. During the Fenton reaction process, the introduction of additional light irradiation leads to the production of more •OH radicals, which benefits from the synergistic effect between the photocatalysis and Fenton reactions [16,17,18,19]. However, the traditional photocatalysis and photo-Fenton-like catalytic processes are active only under the irradiation of ultraviolet (UV) light, which accounts for merely 5% of sunlight, and therefore their practical applications are limited. The development of visible light-driven photocatalysts and photo-Fenton-like catalysts is necessary for their applications in the wastewater treatment.

BiFeO_3_ is one of the important perovskite-type oxides with a narrow band gap of 2.1–2.5 eV, which is suitable for the response of visible light. BiFeO_3_ is shown to be a promising photocatalyst for dye degradation, as well as water splitting under visible-light irradiation [20,21,22,23,24,25,26,27]. Furthermore, BiFeO_3_ also exhibits favorable visible-light-driven photo-Fenton degradation activity [28,29]. Unfortunately, BiFeO_3_ still exhibits a high recombination rate of photoproduced electron-hole pairs, and thus its photocatalytic and photo-Fenton catalytic activity is limited. Until now, many strategies have been developed to enhance the separation rate of photogenerated charges, such as doping with other elements, decoration with noble metals, and construction of heterojunction composites [30,31,32,33,34]. Especially, coupling BiFeO_3_ with an appropriate semiconductor to form the Z-scheme heterojunction was reported to be an efficient and promising strategy, to suppress the recombination of photogenerated charges, and then to enhance its catalytic activity [35,36,37].

Low-dimension nanomaterials like graphene, carbon nanotubes, carbon quantum dots (CQDs), SiC nanowires, and Ag_2_S nanoparticles manifest many attractive properties, and they offer great potential applications for bioimaging, energy conversion, optoelectronic devices, wave absorption, and sensors [38,39,40,41,42,43,44,45,46,47,48,49,50,51]. Moreover, these nanomaterials have been widely used as modifiers or cocatalysts to improve the photocatalytic performances of semiconductors [52,53,54,55,56]. Among them, Ag_2_S is a narrow band-gap semiconductor, and they can absorb visible and near-infrared light. This makes it an important visible-light-driven photocatalytic material [57,58]. Particularly, Ag_2_S has been most frequently used as an ideal co-catalyst, combined with other semiconductors, to form heterojunction composites with improved catalytic activity [59,60,61,62]. Moreover, the construction of heterostructures can significantly decrease the photocorrosion of Ag_2_S. It is noted that bare Ag_2_S could easily undergo photocorrosion, due to the reduction of the lattice Ag^+^ into metal Ag, caused by photogenerated electrons. By comparing the energy band position and bandgap of Ag_2_S with those of BiFeO_3_, the two semiconductors are expected to be coupled together to form excellent Z-scheme Ag_2_S/BiFeO_3_ heterojunction composites. However, no work has been found that is concerned with the visible-light-driven photocatalytic and photo-Fenton catalytic activities of Z-scheme Ag_2_S/BiFeO_3_ composites.

In this work, Ag_2_S nanoparticles were decorated on the surfaces of polyhedral BiFeO_3_ particles to produce Z-scheme Ag_2_S/BiFeO_3_ heterojunction composites via a co-precipitation method. The photocatalytic and photo-Fenton catalytic degradation activities of the Ag_2_S/BiFeO_3_ composites was systematically investigated by degrading methyl orange (MO), using visible light as the light source. The underlying catalytic mechanism of the composites was proposed.

## 2. Theoretical Methods

### 2.1. Preparation of the Ag_2_S Nanoparticles

For the preparation of Ag_2_S, 2 mmol of AgNO_3_ was introduced into 30 mL deionized water, and magnetically stirred to obtain a uniform solution. Exactly 1 mmol of Na_2_S was dissolved in 20 mL deionized water, and the resulting Na_2_S solution was then added into the AgNO_3_ solution (above). The mixture was subjected to vigorous magnetic stirring for 5 h. During this process, a black suspension was obtained. The black product was collected by centrifugation. After washing several times using deionized water and then drying at 60 °C for 6 h in a vacuum oven, the final Ag_2_S nanoparticles were obtained.

### 2.2. Preparation of BiFeO_3_ Polyhedra

For the synthesis of BiFeO_3_, 5 mmol of Bi(NO_3_)_3_·5H_2_O and 5 mmol of Fe(NO_3_)_3_·9H_2_O were added into 20 mL of 25% (*v*/*v*) dilute nitric acid solution under magnetic stirring. Exactly 60 mL of 4.5 mol·L^−1^ KOH solution was dropped into the above solution. After magnetic stirring and ultrasonic treatment, a yellow suspension was obtained. Then, the suspension was transferred into a Teflon-lined stainless steel autoclave (capacity: 100 mL), and heated at 200 °C for 9 h. After that, the product was collected and washed with deionized water. After drying at 80 °C for 12 h, the final product of the BiFeO_3_ polyhedra was obtained.

### 2.3. Fabrication of the Ag_2_S/BiFeO_3_ Composites

For the fabrication of the Ag_2_S/BiFeO_3_ composites, 0.1 g of BiFeO_3_ particles and a certain amount of AgNO_3_ were added into 30 mL of deionized water under vigorous stirring, to achieve a uniform mixture. Exactly 20 mL Na_2_S solution (possessing a certain concentration) was slowly added into the above suspension, which was then subjected to magnetic stirring for 5 h. In this process, the color of the mixture changed from brownish into black, indicative of the successful assembly of Ag_2_S nanoparticles on the surface of the BiFeO_3_ polyhedral particles. After washing and drying, as in the procedure described above, the final Ag_2_S/BiFeO_3_ composite was obtained. By adjusting the amounts of AgNO_3_ and Na_2_S, a series of Ag_2_S/BiFeO_3_ composites with different Ag_2_S mass fractions (5%, 10%, 15%, and 20%) in the composites were fabricated. The corresponding composites were named 5% Ag_2_S/BiFeO_3_, 10% Ag_2_S/BiFeO_3_, 15% Ag_2_S/BiFeO_3_, and 20% Ag_2_S/BiFeO_3_.

### 2.4. The Photo-Fenton Catalytic Activity Test

To investigate the photo-Fenton catalytic activities of the samples, MO was used as the model pollutant, and the visible light (300 W xenon lamp with a 420 nm cut-off filter) was used as the light source. A certain amount of the catalyst was dispersed into the MO solution (100 mL) in the dark under magnetic stirring for 30 min. During this process, the absorption/desorption equilibrium between the catalyst and the dye was achieved. After that, an amount of H_2_O_2_ was added into the reaction solution, and then irradiated by visible light. During the reaction, 2.5 mL of the reaction solution was sampled at a given time interval, and centrifuged to remove the catalyst. The residual MO concentration was obtained by measuring the absorbance of the reaction solution at λ_MO_ = 464 nm. The effects of catalyst dosage, H_2_O_2_ concentration, and MO concentration on the degradation efficiency of MO were investigated, based on the above experimental process. The photocatalytic activity of the samples was evaluated under the same conditions without the addition of H_2_O_2_. Ethanol (10% (*v*/*v*)), ammonium oxalate (AO, 2 mmol·L^−1^) and benzoquinone (BQ, 1 mmol·L^−1^) were separately added into the reaction solution, with the aim of examining the active species in the catalytic process.

### 2.5. Photoelectrochemical Measurement

An electrochemical workstation (CHI 660C, Shanghai Chenhua Instrument Co. Ltd, Shanghai, China) with a three-electrode system was used for the photoelectrochemical measurements [63]. In the three-electrode system, a platinum foil acted as the counter-electrode, and a standard calomel electrode was used as the reference electrode. For the preparation of the working electrode, stoichiometric amounts of photocatalyst (15 mg), carbon-black (0.75 mg), and polyvinylidene fluoride (PVDF, 0.75 mg) were uniformly dispersed in 1 mL 1-methyl-2-pyrrolidione (NMP) to obtain a slurry mixture. The slurry mixture was coated onto a fluoride-doped tin oxide glass electrode (effective area: 1.0 × 1.0 cm^2^), and dried at 60 °C for 5 h. During the photoelectrochemical measurements, a 300 W xenon lamp with a 420 nm cut-off filter was used as the light source, and 1 mol L^−1^ Na_2_SO_4_ aqueous solution was used as the electrolyte. A 0.2 V bias potential was applied for the testing of photocurrent–time (*I*-*t*) curves. The electrochemical impedance spectroscopy (EIS) spectra were recorded by applying a sinusoidal voltage pulse (amplitude: 5 mV; frequency range: 10^−2^–10^5^ Hz).

### 2.6. Hydroxyl Radical Measurement

The •OH radicals generated on the irradiated samples were measured by fluorimetry. To achieve this aim, terephthalic acid (TA) as an •OH scavenger [64]. The measurement procedure was as follows: TA was added into an NaOH solution with a concentration of 1.0 mmol·L^−1^, to obtain a 0.25 mmol·L^−1^ TA solution. Exactly 60 mg of the catalyst was added into 100 mL of TA solution. The mixture was magnetically stirred for several minutes to ensure the uniform dispersion of the catalyst. Subsequently, H_2_O_2_ was added into the reaction solution (2.0 mmol·L^−1^) and then irradiated by a 300 W xenon lamp with a 420 nm cut-off filter. At a given time interval, 2.5 mL of the reaction solution was sampled to measure its photoluminescence (PL) spectrum (the catalyst was removed by centrifugation). A fluorescence spectrophotometer was used to measure the PL spectrum of the reaction solution (excitation wavelength: 315 nm). The •OH radicals produced during the photocatalytic process were detected under the same conditions without the introduction of H_2_O_2_.

### 2.7. Characterization

X-ray powder diffraction (XRD) was employed to investigate the phase purity of the samples on a D8 Advance X-ray diffractometer (Bruker AXS, Karlsruhe, Germany). The morphologies and microstructures of the products were observed by field-emission scanning electron microscopy (SEM) and field-emission transmission electron microscopy (TEM). The SEM investigation was performed on a JSM-6701F scanning electron microscope (JEOL Ltd., Tokyo, Japan), and the TEM observation was carried out on a JEM-1200EX transmission electron microscope (JEOL Ltd., Tokyo, Japan). X-ray photoelectron spectroscopy (XPS) was used to record the chemical states of the elements on a PHI-5702 multi-functional X-ray photoelectron spectrometer (Physical Electronics, Chanhassen, MN, USA). The ultraviolet–visible (UV-Vis) diffuse reflectance spectra of the samples were tested by using a TU-1901 double beam UV-Vis spectrophotometer (Beijing Purkinje General Instrument Co. Ltd., Beijing, China) with BaSO_4_ as a reference. An RF-6000 fluorescence spectrophotometer (Shimadzu, Kyoto, Japan) was available to record the PL spectra of the samples (excitation wavelength: ~350 nm).

## 3. Results and Discussion

### 3.1. XRD Analysis

**Figure 1:**
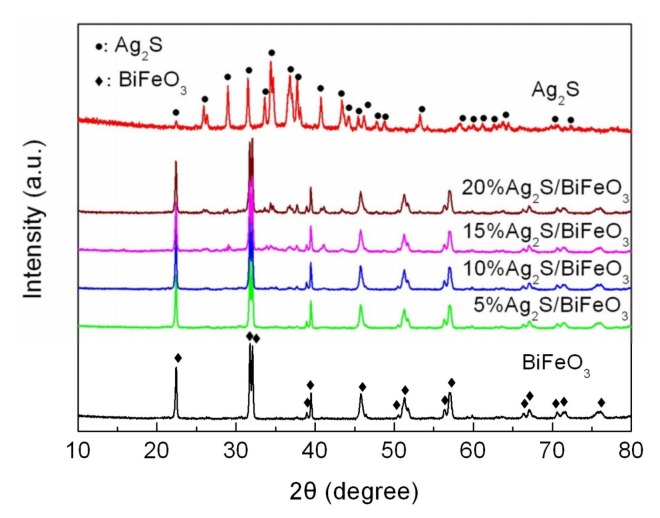
X-ray powder diffraction (XRD) patterns of BiFeO_3_, Ag_2_S, and Ag_2_S/BiFeO_3_ composites.

Figure 1 shows the XRD patterns of the BiFeO_3_, Ag_2_S and Ag_2_S/BiFeO_3_ composites. For the bare BiFeO_3_ and Ag_2_S samples, their diffraction peaks could be completely indexed in terms of the rhombohedral structure of BiFeO_3_ (PDF card no. 74-2016) and the monoclinic structure of Ag_2_S (PDF card no. 14-0072), respectively. When Ag_2_S is decorated on BiFeO_3_, the diffraction peaks of BiFeO_3_ undergo no obvious change, suggesting that BiFeO_3_ maintains a rhombohedral structure. Meanwhile, weak characteristic diffraction peaks of Ag_2_S are observed in the composites, indicating the decoration of Ag_2_S on BiFeO_3_. In addition, no other impurity phases are detected in the composites.

### 3.2. Optical Absorption Properties

**Figure 2:**
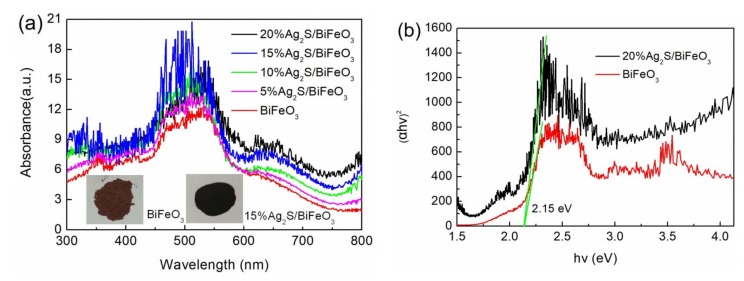
(**a**) UV-Vis absorption spectra of BiFeO_3_ and Ag_2_S/BiFeO_3_ composites. The insets show the digital images of BiFeO_3_ and 15% Ag_2_S/BiFeO_3_. (**b**) The corresponding Tauc plots of (α*hν*)^2^ vs *hν* for BiFeO_3_ and 20% Ag_2_S/BiFeO_3_.

Figure 2a shows the absorption spectra of the BiFeO_3_ and Ag_2_S/BiFeO_3_ composites transformed from the UV-Vis spectra, using the Kubelka–Munk (K-M) theory. It is worth noting that, with the increase of Ag_2_S content, the composites exhibit a gradually increasing light absorption over the whole wavelength range, which is mainly due to the strong light absorption of Ag_2_S in the UV-Vis light region. The digital images of the samples inserted in Figure 2a show that the color of bare BiFeO_3_ is brown, and the 15% Ag_2_S/BiFeO_3_ composite manifests a black color. The deepening of the apparent color for the composite further confirms its enhanced visible-light absorption. To obtain the energy bandgap of the samples, the plots of (α *hν*)^2^ vs *hν* using the Tauc relation are carried out, where α is defined as the K-M absorption coefficient, and *hν* is the incident photon energy. As depicted in Figure 2b, the bandgap (*E*_g_) of bare BiFeO_3_ is estimated to be 2.15 eV by extrapolating the linear portion of the plot to the *hν* axis. A similar *E*_g_ of BiFeO_3_ in the 20% Ag_2_S/BiFeO_3_ composite is obtained, suggesting that the band structure of BiFeO_3_ undergoes no detectable change after the decoration of Ag_2_S. In addition, based on its bandgap energy, the valence band (VB) and the conduction band (CB) potentials of BiFeO_3_ can be obtained by using the following relations (Equations (1) and (2)):

*E*_VB_ = *X* − *E*^e^ + 0.5 *E*_g_ (1)

*E*_CB_ = *X* − *E*^e^ − 0.5 *E*_g_ (2)

In the above relations, *X* is the absolute electronegativity of BiFeO_3_, and it is estimated to be 5.93 eV, based on the data reported in the literature [65]. *E*^e^ is defined as the energy of free electrons on the hydrogen scale (~4.5 eV). The VB and CB potentials of BiFeO_3_ were found to be +2.5 and +0.35 V vs normal hydrogen electrode (NHE), respectively.

### 3.3. XPS Analysis

**Figure 3:**
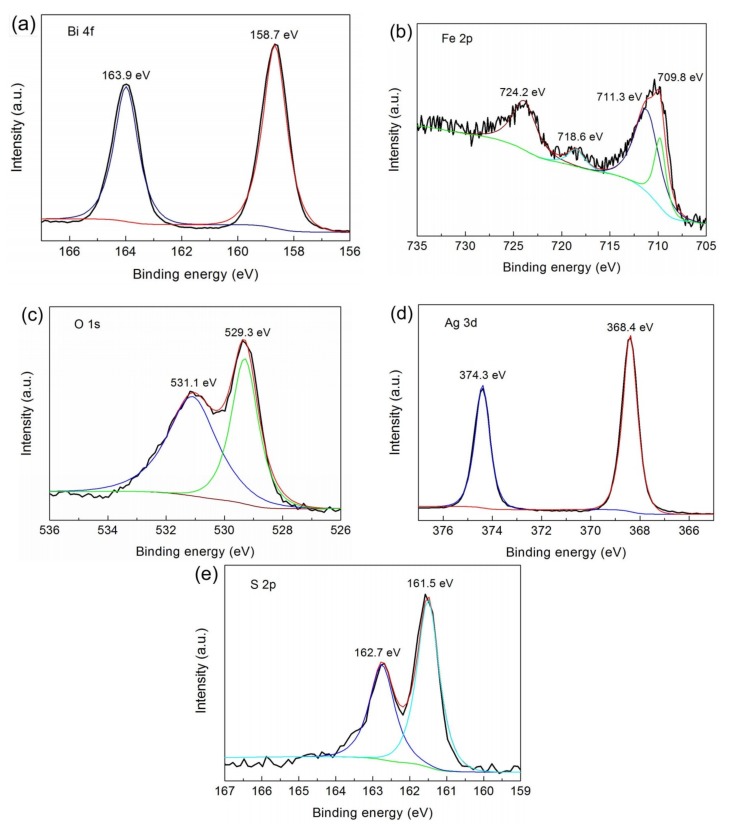
High-resolution X-ray photoelectron spectroscopy (XPS) spectra of the 15% Ag_2_S/BiFeO_3_ composite. (**a**) Bi 4f; (**b**) Fe 2p; (**c**) O 1s; (**d**) Ag 3d; (**e**) S 2p.

To investigate the surface chemical states of 15% Ag_2_S/BiFeO_3_, XPS detection was carried out. Figure 3a–e show the high-resolution XPS spectra of Bi 4f, Fe 2p, O 1s, Ag 3d, and S 2p, respectively. From Figure 3a, the Bi 4f_5/2_ and Bi 4f_7/2_ binding energy peaks were found at 163.9 and 158.7 eV, respectively, indicating the existence of Bi^3+^ [21]. Figure 3b depicts the Fe 2p spectrum, which displays two separate peaks at 724.2 (Fe 2p_1/2_) and 710.1 eV (Fe 2p_3/2_), respectively. The Fe 2p_3/2_ binding energy can be divided into two peaks at 711.3 and 709.8 eV, corresponding to Fe^3+^ and Fe^2+^, respectively [21,66,67]. The weak peak detected at 718.6 eV is characterized as the satellite peak for Fe^3+^. Figure 3c presents the O 1s spectrum. By fitting the O 1s binding energy, two peaks at 529.3 and 531.1 eV are found. The signal at 529.3 eV is assigned to the lattice oxygen, while the peak at 531.1 eV is mainly attributed to the surface vacancies and the chemisorbed oxygen [21,62]. On the Ag 3d spectrum (Figure 3d), the peak at 368.4 eV is assigned to Ag 3d_5/2_, and the peak at 374.3 eV is attributed to Ag 3d_3/2_ of Ag^+^ [68]. In the spectrum of S 2p (Figure 3e), the signal of S 2p can be deconvoluted into two peaks at 162.7 and 161.5 eV, which are caused by the S 2p_1/2_ and S 2p_3/2_, respectively [68].

### 3.4. Morphology Observations

**Figure 4:**
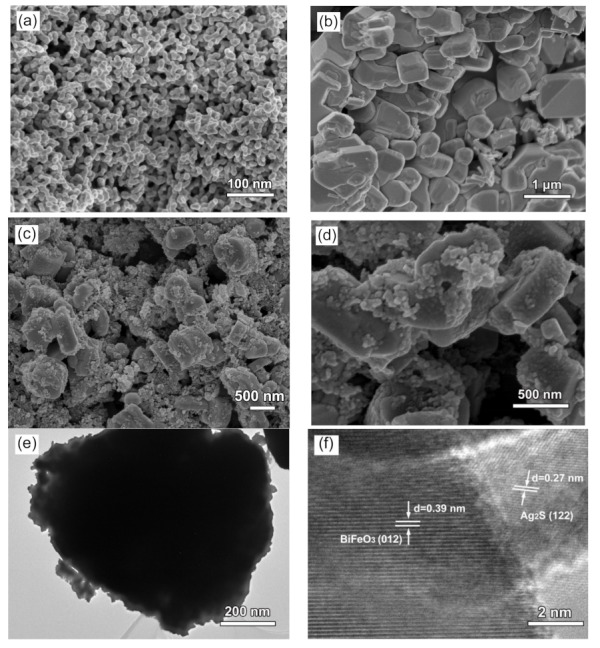
(**a**–**d**) Scanning electron microscopy (SEM) images of Ag_2_S nanoparticles, BiFeO_3_ particles and 15% Ag_2_S/BiFeO_3_, respectively. (**e**) and (**f**) Transmission electron microscopy (TEM) image and high-resolution (HR)TEM image of 15% Ag_2_S/BiFeO_3_, respectively.

**Figure 5:**
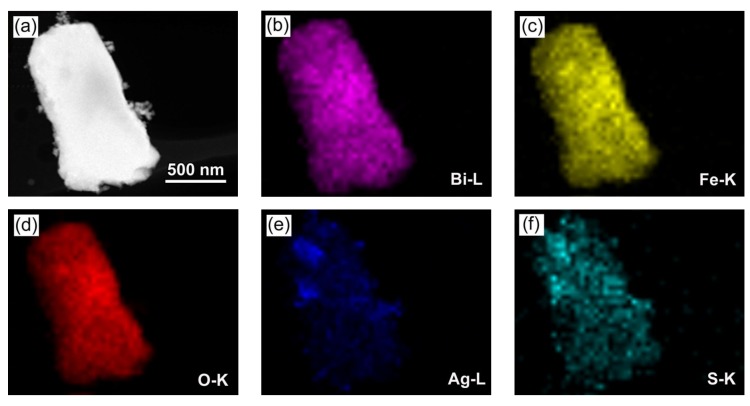
(**a**) Dark-field scanning TEM (DF-STEM) image of 15% Ag_2_S/BiFeO_3_. (**b**–**f**) The energy-dispersive X-ray elemental mapping images of the region shown in (a).

Figure 4a–d shows the SEM images of Ag_2_S, BiFeO_3_, and 15% Ag_2_S/BiFeO_3_. It is seen that Ag_2_S exhibits spherical-like shapes with diameters of 30–50 nm. The BiFeO_3_ sample is mainly composed of polyhedral-like particles with edge lengths of 500–800 nm, and the particles have smooth surfaces. From the SEM image of the 15% Ag_2_S/BiFeO_3_ composite, small Ag_2_S nanoparticles are clearly observed to be attached onto the surface of the BiFeO_3_ polyhedral-like particles. Figure 4e presents the TEM image of the 15% Ag_2_S/BiFeO_3_ composite, further confirming the decoration of the Ag_2_S nanoparticles on the polyhedral BiFeO_3_ particles. The high-resolution TEM (HRTEM) image obtained from the interface of the two phases in the 15% Ag_2_S/BiFeO_3_ composite is shown in Figure 4f, revealing two distinct sets of lattice fringes. The interplanar spacing of ~0.39 nm corresponds to the (012) plane of BiFeO_3_, and the interplanar spacing of ~0.27 nm corresponds to the (122) plane of Ag_2_S. This indicates the intimate contact between Ag_2_S and BiFeO_3_ to form a Ag_2_S/BiFeO_3_ heterojunction structure. To further investigate the distribution of Ag_2_S on the surface of BiFeO_3_, the elemental mapping observation of 15% Ag_2_S/BiFeO_3_ was performed, as shown in Figure 5. Figure 5a depicts the dark-field scanning TEM (DF-STEM) image of the composite, and Figure 5b–f display the corresponding elemental maps that are obtained according to the procedure described in the literature [69]. It was found that large-sized polyhedra show an elemental distribution of Bi, Fe and O, whereas the attached small particles contain the elements Ag and S. The result suggests that the Ag_2_S nanoparticles are uniformly anchored onto the surface of the polyhedral BiFeO_3_ particles.

### 3.5. Photo-Fenton Catalytic and Photocatalytic Performances

**Figure 6:**
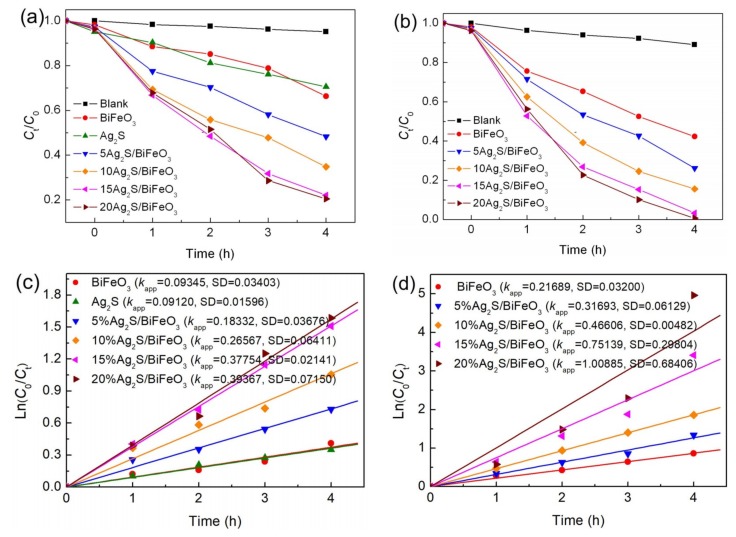
(**a**) Photocatalytic activities of Ag_2_S, BiFeO_3_, and Ag_2_S/BiFeO_3_ composites toward the degradation of MO under visible-light irradiation, along with the blank and adsorption experiment results. (**b**) Photo-Fenton catalytic activities of BiFeO_3_ and Ag_2_S/BiFeO_3_ composites toward the degradation of MO under visible-light irradiation, and in the presence of H_2_O_2_, along with blank and adsorption experimental results. (**c**) Plots of Ln(*C*_t_/*C*_0_) vs reaction time *t* for the photocatalytic degradation of MO over the samples. (**d**) Plots of Ln(*C*_t_/*C*_0_) vs reaction time *t* for the photo-Fenton catalytic degradation of MO over the samples.

MO was used as the model pollutant to evaluate the visible-light-driven photocatalytic activities of the samples, as shown in Figure 6a. The direct photolysis of the dye without the photocatalyst, and the dye adsorption on the photocatalyst were also investigated, which reveals that the dye MO has a good stability after a short period of visible-light irradiation; the absorption of the dye on the photocatalyst is very small. After 4 h of photocatalysis, the introduction of BiFeO_3_ and Ag_2_S leads to MO degradation percentages of ~33% and ~30%, respectively. This implies a relatively weak photocatalytic activity from bare BiFeO_3_ and Ag_2_S. When Ag_2_S is decorated on BiFeO_3_, the formed Ag_2_S/BiFeO_3_ composites exhibit obviously improved photocatalytic activities. With an increase in the Ag_2_S content, an optimal composite sample is observed for 15% Ag_2_S/BiFeO_3_. Further increasing the Ag_2_S content cannot result in an enhanced photocatalytic activity for the composites. This suggests that the construction of the Ag_2_S/BiFeO_3_ heterojunction is critical for the effective enhancement of photocatalytic activity. However, the excessive Ag_2_S loading may result in full coverage by Ag_2_S on the BiFeO_3_ surface, thus decreasing the photon absorption of BiFeO_3_. To further compare the photocatalytic activities of the samples, kinetic analysis of the photocatalytic degradation of MO was carried out. As illustrated in Figure 6c, the plots of Ln(*C*_t_/*C*_0_) vs reaction time *t* presented a good linear behavior, and they can be modeled well by using the first-order kinetic equation Ln(*C*_t_/*C*_0_) = *k*_app_*t*, where *k*_app_ is the apparent first-order reaction rate constant. In every case, the standard deviation (SD) was smaller than 0.07. The obtained values of *k*_app_ and SD are inserted in Figure 6c. From the reaction rate constants, it is concluded that the optimal composite sample, 15% Ag_2_S/BiFeO_3_, has a photocatalytic activity ca. 4.0 and 4.1 times higher than those of bare BiFeO_3_ and Ag_2_S, respectively.

To further examine the photo-Fenton catalytic behavior of the samples, the degradation of MO in the photo-Fenton processes was performed, as shown in Figure 6b. In the absence of the photocatalyst, the degradation of MO is observed to be very small under visible light irradiation and in the presence of H_2_O_2_, indicating minor self-degradation for MO. With the addition of catalysts and H_2_O_2_ under visible light irradiation, dye degradation is significantly enhanced, to be much higher than that in the bare photocatalytic process. The photo-Fenton catalytic activities of the samples are in the same orders as their photocatalytic activities. The kinetic plots for the photo-Fenton catalytic degradation of MO are shown in Figure 6d. The derived reaction rate constants reveal that the 15% Ag_2_S/BiFeO_3_ composite exhibits a photo-Fenton catalytic activity ca. 3.5 times higher than that of bare BiFeO_3_.

**Figure 7:**
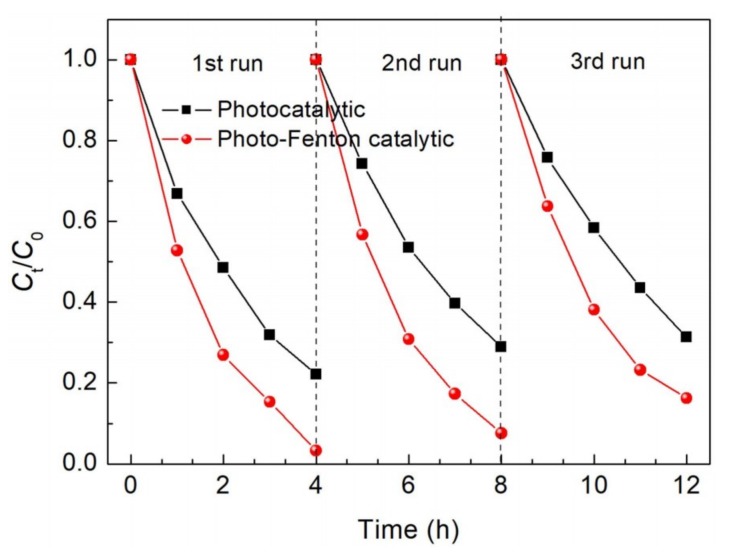
Photocatalytic and photo-Fenton catalytic recyclabilities of the 15% Ag_2_S/BiFeO_3_ composite for the degradation of MO under visible-light irradiation.

Figure 7 shows the degradation percentage of MO over the 15% Ag_2_S/BiFeO_3_ composite during three successive photocatalytic or photo-Fenton catalytic processes. It is seen that the catalytic activity of the composite does not undergo a significant decrease. After three successive recycles, the photocatalytic and photo-Fenton catalytic degradation percentages of MO still reach ~70% and ~84%, respectively.

**Figure 8:**
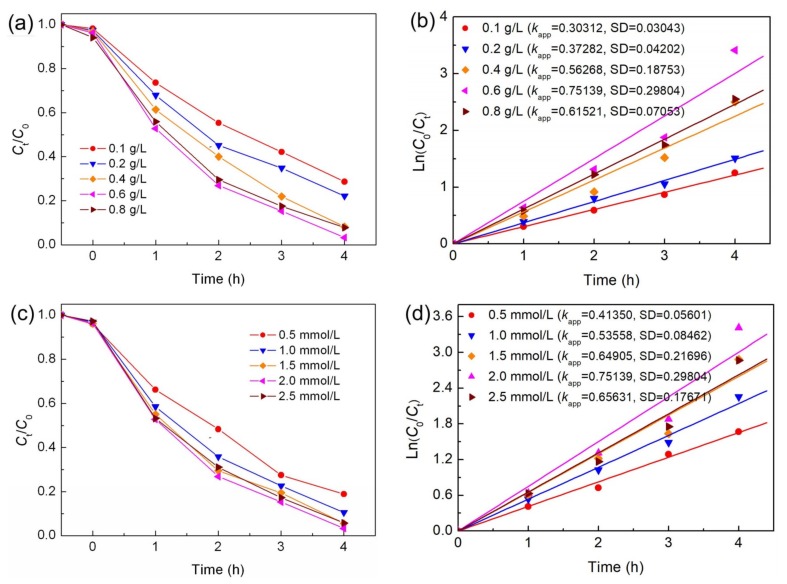

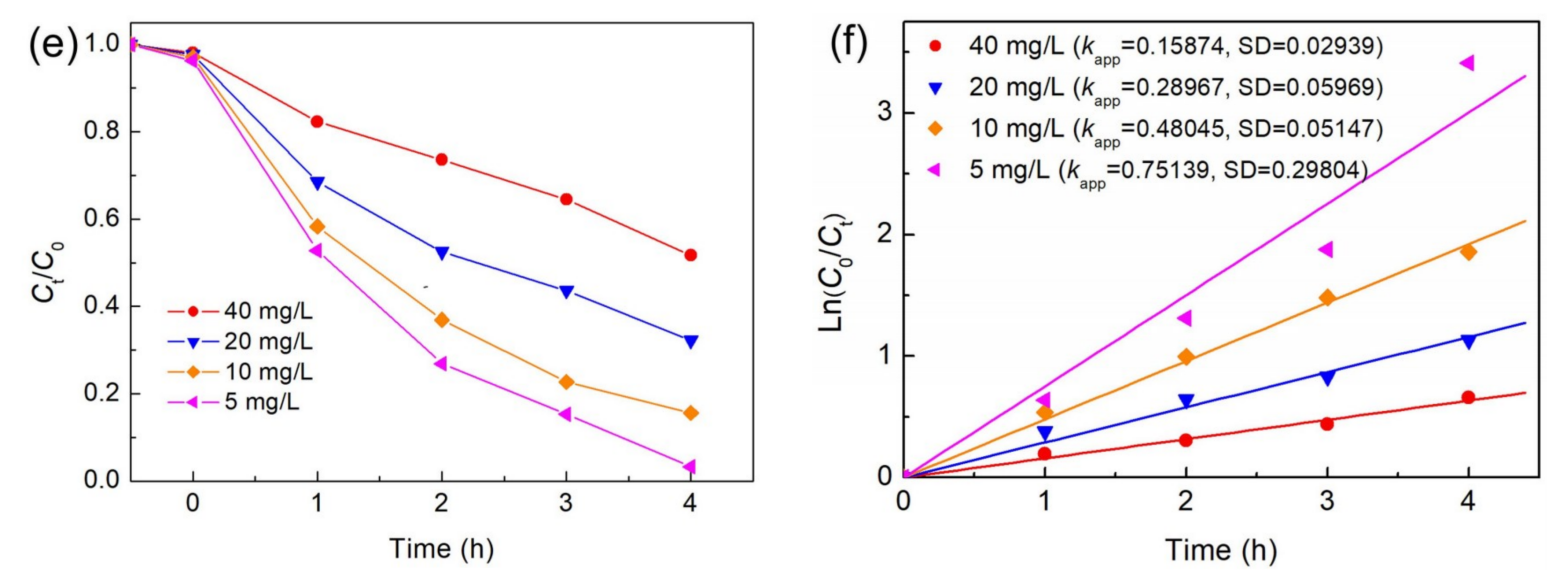
Effects of the photocatalyst (15% Ag_2_S/BiFeO_3_) dosage (**a**) and (**b**), H_2_O_2_ content (**c**) and (**d**), and MO concentration (**e**) and (**f**) on the photo-Fenton catalytic degradation of MO, respectively.

Choosing 15% Ag_2_S/BiFeO_3_ as the photocatalyst, further photo-Fenton degradation experiments were carried out, aimed at investigating the effects of photocatalyst dosage, H_2_O_2_ content, and dye concentration on dye degradation. Figure 8a shows the effect of catalyst dosage on the degradation of MO. The corresponding kinetic plots are presented in Figure 8b. The degradation percentage of MO increases gradually with an increase in the catalyst loading from 0.1 to 0.6 g·L^−1^. However, when the catalyst loading exceeds the optical value (0.6 g·L^−1^), the catalytic efficiency starts to decrease. Generally, increasing the catalyst loading can provide more active sites for H_2_O_2_ activation to produce more •OH radicals, thus accelerating the degradation of the dye. Nevertheless, excessive addition of the catalyst will enhance the light screening effect, and reduce light absorption, thus resulting in a decrease in the catalytic efficiency.

The effect of H_2_O_2_ concentration on the degradation of MO is shown in Figure 8c. As observed, the degradation percentage of MO increases from ~81% to ~97%, with an increase in the H_2_O_2_ concentration from 0.5 to 2 mmol·L^−1^, which is due to the fact that more •OH radicals can be produced by increasing the H_2_O_2_ concentration. However, when the concentration of H_2_O_2_ reaches 2.5 mmol·L^−1^, the MO degradation percentage exhibits a decreasing trend. This phenomenon is mainly attributed to the consumption of •OH radicals by the reaction of additional H_2_O_2_ with •OH [15]. The corresponding kinetic plots and derived reaction rate constants (Figure 8d) further confirm the effect of H_2_O_2_ concentration on the degradation of MO.

The effect of MO concentration on its degradation is shown in Figure 8e. The corresponding kinetic plots are presented in Figure 8f. It is seen that the degradation percentage of MO decreases with increasing the initial MO concentration, which is due to the decreased light transparency. In spite of this, the 15% Ag_2_S/BiFeO_3_ composite still photo-Fenton-catalyzes a 50% rate of removal of the dye at a high MO concentration of 40 mg·L^−1^. This implies that the present Ag_2_S/BiFeO_3_ composite photocatalyst could offer a practical application in the treatment of high-concentration dye wastewater. From an application viewpoint, the stability of the catalyst is another important factor to be considered besides its catalytic activity.

### 3.6. Photogenerated Charge Behavior

**Figure 9:**
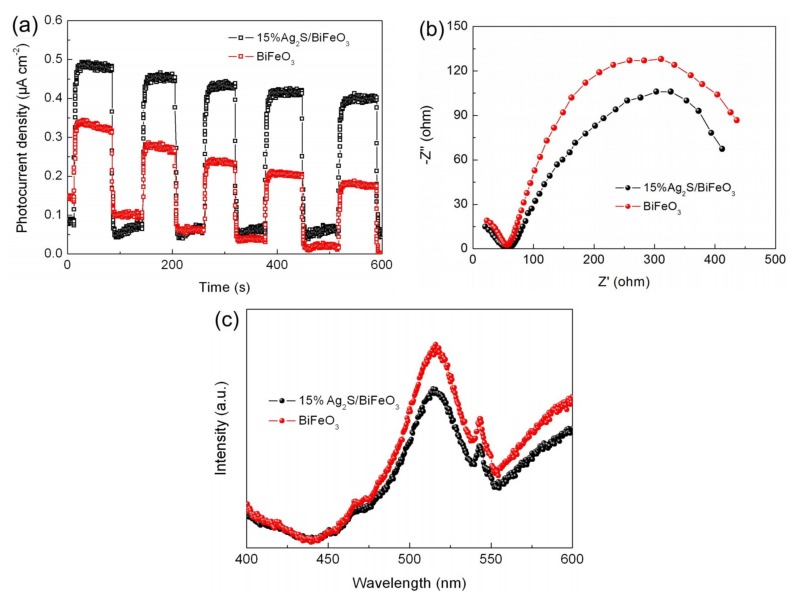
Transient photocurrent response curves under intermittent irradiation with visible light (**a**), Nyquist plots of the electrochemical impedance spectroscopy (EIS) spectra under visible-light irradiation (**b**), and PL spectra (**c**) of BiFeO_3_, and 15% Ag_2_S/BiFeO_3_.

It is known that photocatalytic and photo-Fenton catalytic activities are related to the performance of the photogenerated charges. Photoelectrochemical and PL measurements were performed to investigate the photoinduced charge behaviors of the samples [70,71]. Figure 9a shows the photocurrent response profiles of BiFeO_3_ and 15% Ag_2_S/BiFeO_3_. Compared with bare BiFeO_3_, the 15% Ag_2_S/BiFeO_3_ composite exhibits an enhanced photocurrent density, suggesting that the decoration of Ag_2_S nanoparticles results in an enhanced separation of photogenerated e^−^-h^+^ pairs. The EIS spectra of BiFeO_3_ and 15% Ag_2_S/BiFeO_3_ are presented in Figure 9b. In the EIS spectra, the arc radius for 15% Ag_2_S/BiFeO_3_ is smaller than that for bare BiFeO_3_, which indicates the low interfacial resistance of the composite. Figure 9c displays the PL spectra of BiFeO_3_ and 15% Ag_2_S/BiFeO_3_. A steady emission peak is detected at ~520 nm, which is mainly due to the recombination of the photogenerated charges. The 15% Ag_2_S/BiFeO_3_ composite possesses a relatively weak PL emission peak in comparison to bare BiFeO_3_, indicating that the recombination of photoinduced charges is suppressed in the composite. The above results reveal that the photogenerated electrons and holes of BiFeO_3_ are successfully separated and migrated by the decorations of the Ag_2_S nanoparticles.

### 3.7. Active Species Detection

**Figure 10:**
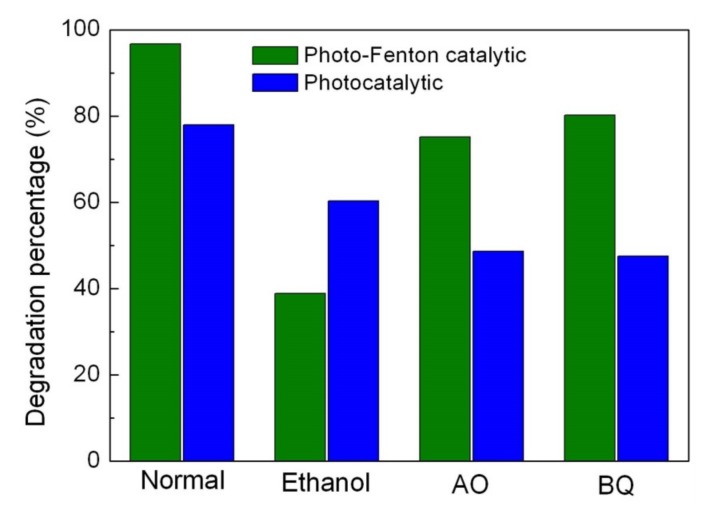
Effects of ethanol, benzoquinone (BQ) and ammonium oxalate (AO) on the photocatalytic and photo-Fenton catalytic degradation rates of MO over 15% Ag_2_S/BiFeO_3_ under visible-light irradiation.

To unveil the active species that are responsible for the photocatalytic and photo-Fenton catalytic degradation of MO over the Ag_2_S/BiFeO_3_ composites, the active species trapping experiments were performed. To achieve this aim, ethanol was used as the •OH scavenger, AO as the h^+^ scavenger, and BQ as the •O_2_^−^ scavenger [72], and the results are shown in Figure 10. During the photocatalytic process, the introduction of AO leads to an obvious reduction of dye degradation, and a similar suppression effect is detected after the addition of BQ. When ethanol is added, the degradation efficiency of the dye is slightly decreased. The above results suggest that h^+^ and •O_2_^−^ are considered to be the major reactive species, while •OH plays a relatively small role in the photocatalytic degradation of MO. In the photo-Fenton catalytic reaction, ethanol can greatly inhibit the degradation of MO. Compared with ethanol, AO and BQ exhibit relatively weak inhibition effects on dye degradation. This indicates that the photo-Fenton catalytic degradation of MO over the Ag_2_S/BiFeO_3_ composite is primarily induced by the attack of h^+^, •O_2_^−^, and •OH, and •OH plays a relatively large role in the photo-Fenton catalytic reaction.

**Figure 11:**
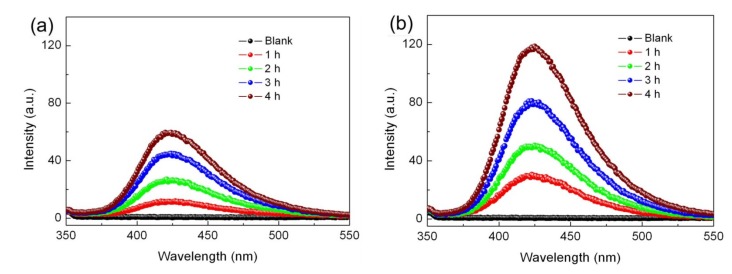
Time-dependent PL spectra of the terephthalic acid (TA) solution after (**a**) photocatalytic and (**b**) photo-Fenton reactions over 15% Ag_2_S/BiFeO_3_.

Figure 11a,b shows the time-dependent PL spectra of the TPA solution over the 15% Ag_2_S/BiFeO_3_ composite in the photocatalytic and the photo-Fenton catalytic processes, respectively. During the photocatalytic and photo-Fenton catalytic processes, an obvious PL signal is detected at around 429 nm, and its intensity exhibits an increasing trend with the irradiation time, suggesting that the production of the •OH radicals during the photocatalytic and photo-Fenton catalytic processes. However, the PL signal becomes more intense in the photo-Fenton process, which indicates the generation of more •OH radicals. This result is consistent with the active species-trapping experiments.

### 3.8. Photocatalytic and Photo-Fenton Mechanisms

**Figure 12:**
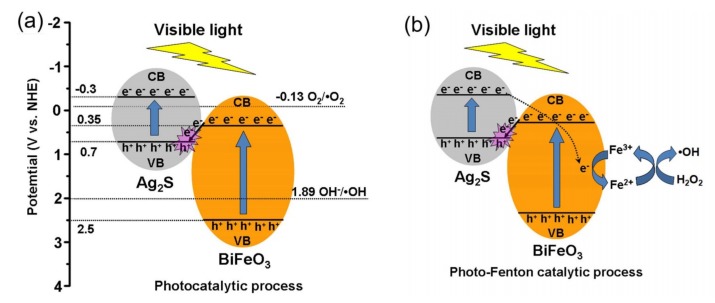
Schematic illustration of the degradation mechanism of MO over the Ag_2_S/BiFeO_3_ composites. (**a**) The photocatalytic process; (**b**) the photo-Fenton catalytic process.

The photocatalytic mechanism of the Ag_2_S/BiFeO_3_ composites are schematically shown in Figure 12a. Based on the previous report, the CB/VB edge potentials of Ag_2_S are estimated to be −0.3/+0.7 V vs NHE [73]. The CB/VB edge potentials of BiFeO_3_ are obtained as +0.35/+2.5 V vs NHE, respectively. This suggests that BiFeO_3_ and Ag_2_S can be coupled together to form a promising Z-scheme composite photocatalysts, due to their well-matched overlapping band-structures. Under illumination from visible light, both BiFeO_3_ and Ag_2_S are excited to generate electrons in the CB, and holes in the VB. For bare BiFeO_3_ and Ag_2_S, most of the photogenerated charges tend to be recombined, and only a few of them take part in the photocatalytic reaction. In contrast, in the Ag_2_S/BiFeO_3_ heterojunction composites, the photoexcited electrons in the CB of BiFeO_3_ will migrate to Ag_2_S and combine with the photoexcited holes in the VB of Ag_2_S. This charge migration and combination process remarkably promote the separation of the photogenerated electrons, and holes in BiFeO_3_ and Ag_2_S. As a result, more photoinduced holes in BiFeO_3_ and photoinduced electrons in Ag_2_S are able to take part in the photocatalytic reaction. This is the dominant mechanism, resulting in enhanced photocatalytic activity for the Ag_2_S/BiFeO_3_ composites. Compared to the redox potential of O_2_/•O_2_^−^ (−0.13 V vs NHE) [21], the sufficiently negative CB potential of Ag_2_S indicates that the photoexcited electrons in Ag_2_S can reduce O_2_ to generate •O_2_^−^. On the other hand, the photogenerated holes in the VB of BiFeO_3_ possess enough oxidation ability to generate •OH (*E*^0^(OH^−^/•OH) = +1.99 V vs NHE) [74]. Under visible-light irradiation, and in the presence of H_2_O_2_, photocatalytic and Fenton reactions simultaneously take place, and moreover, the two catalytic processes exhibit an important synergistic effect, as schematically shown in Figure 12b. In the photo-Fenton catalytic process, Fe^2+^ on the surface of BiFeO_3_ can react with H_2_O_2_ to generate •OH and Fe^3+^. Then, Fe^3+^ will be reduced into Fe^2+^ by the photogenerated electrons. In this cyclic reaction, more •OH radicals are being produced, but the consumption of the photogenerated electrons leads to the decreased generation of •O_2_^−^ radicals. This could be the reason that •OH plays an important role in the degradation of the dye during the photo-Fenton catalytic process. The main photo-Fenton reaction process can be briefly described by Equations (3)–(9):

BiFeO_3_ + *hν* → BiFeO_3_(e^−^ + h^+^) (3)

Ag_2_S + *hν* → Ag_2_S (e^−^ + h^+^) (4)

h^+^(BiFeO_3_) + OH^−^ → •OH (5)

e^−^(Ag_2_S) + O_2_ → •O_2_^−^ (6)

Fe^2+^ + H_2_O_2_ → Fe^3+^ + •OH + OH^−^ (7)

Fe^3+^ + e^−^(Ag_2_S) → Fe^2+^ (8)

•OH, h^+^, •O_2_^−^ + MO → degradation products (9)

## 4. Conclusions

A series of Z-scheme Ag_2_S/BiFeO_3_ heterojunction composites have been constructed by assembling Ag_2_S nanoparticles on the surface of the BiFeO_3_ polyhedra. It is demonstrated that the Ag_2_S/BiFeO_3_ composites can be employed as a promising catalyst for the photocatalytic and photo-Fenton catalytic decomposition of MO under visible-light irradiation. Compared to the bare photocatalytic process, the synergistic effect of photo-Fenton catalysis in the presence of H_2_O_2_ leads to a higher degradation efficiency of the dye. The optimum composite sample is observed for 15% Ag_2_S/BiFeO_3_, which photo-Fenton-catalyzes ~97% degradation of MO after 4 h of reaction under the optimal conditions. The enhanced catalytic activity of the Z-scheme Ag_2_S/BiFeO_3_ heterojunction composites can be explained as the result of efficient separation of photoexcited e^−^-h^+^ pairs, resulting from the Z-scheme electron transfer.
